# The diagnostic value of angiotensin, g-GT, blood lactate, and renal resistance index combined detection in acute kidney injury after neonatal asphyxia

**DOI:** 10.5937/jomb0-56995

**Published:** 2025-09-05

**Authors:** Xiaoqing Shi, Liying Dai, Ying Wang, Fang Deng

**Affiliations:** 1 Children's Medical Center of Anhui Medical University, Department of Pediatric Nephrology, Hefei, Anhui, China; 2 Anhui Provincial Children's Hospital, Department of Neonatology, Hefei, Anhui, China

**Keywords:** neonate, asphyxia, Acute kidney injury, angiotensin, gamma-glutamyltransferase, blood lactic acid, renal resistive index, novorođenče, asfiksija, akutna povreda bubrega, angiotenzin, gama-glutamiltransferaza, mlečna kiselina u krvi, indeks otpornosti bubrega

## Abstract

To investigate the diagnostic value of angiotensin (Ang), gamma-glutamyltransferase (g-GT), blood lactic acid and renal resistive index (RRI) for acute kidney injury (AKI) after neonatal asphyxia.

## Abstract (sr)

**Uvod:**

Cilj je bio da se ispita dijagnostička vrednost angiotenzina (Ang), gama-glutamiltransferaze (g-GT), mlečne kiseline u krvi i indeksa rezistencije bubrega (RRI) za akutnu povredu bubrega (AKI) posle neonatalne asfiksije.

## Introduction

Neonatal asphyxia is a critical condition that affects neonatal growth and development. Hypoxia and acidosis caused by asphyxia can lead to changes in blood distribution, reduced renal blood flow, and induce acute kidney injury (AKI). Studies have shown that neonatal asphyxia, apart from sepsis and premature birth, is an independent risk factor for neonatal AKI [1] [2]. AKI following neonatal asphyxia increases the risk of both short-term and long-term poor prognosis. Therefore, early diagnosis of AKI after neonatal asphyxia and the implementation of active interventions are crucial for improving neonatal outcomes. Currently, the diagnosis of neonatal AKI mainly relies on serum creatinine (SCr) levels and urine output changes [3] [4]. However, these indicators are significantly influenced by maternal factors, and their level changes may lag behind renal function impairment, resulting in poor sensitivity. Thus, identifying biomarkers and diagnostic methods with higher sensitivity and accuracy has become a key focus of research. Angiotensin (Ang) is an important component of the renin-angiotensin system (RAS) in organ tissues and is involved in regulating renal blood flow and endothelial function [5]. Gamma-glutamyl transferase (γ-GT) is secreted by renal tubular epithelial cells, and elevated levels indicate renal dysfunction. Blood lactate is a metabolic product in tissue hypoxia and is not only related to the severity of neonatal asphyxia but also serves as an important predictor of prognosis in AKI patients. The renal resistance index (RRI) is a non-invasive indicator used to assess renal perfusion, and it has significant value in the early diagnosis of AKI. Currently, there are few studies on the use of these indicators for early diagnosis of AKI after neonatal asphyxia [6] [7]. This study analyzes the changes in blood Ang, blood lactate, RRI, and urinary γ-GT levels in neonates with asphyxia, aiming to provide a reference for the early diagnosis of AKI in clinically affected neonates.

## Materials and methods

### General information

A retrospective analysis was conducted on the clinical data of 120 neonates with asphyxia, with cases included from May 2021 to April 2024. Inclusion criteria: (1) Clinical examination met the diagnostic criteria for mild or severe asphyxia [8]: Mild asphyxia: Apgar score 7 at 1 minute or 5 minutes, with umbilical artery pH <7.2; Severe asphyxia: Apgar score 3 at 1 minute or 5 at 5 minutes, with umbilical artery pH <7.0; (2) Full-term neonates; (3) Blood and urine samples collected within 24 hours after birth; (4) Complete related tests and data.

Exclusion criteria: (1) Neonates who died within 24 hours after birth; (2) Severe congenital diseases; (3) Hemolysis or pathological jaundice; (4) Urinary tract infections caused by other reasons; (5) Missing general data. Among the 120 neonates, 71 were male and 49 were female, with an average gestational age of (39.43±1.21) weeks and average birth weight of (3354.75±341.60) gr. Delivery methods included vaginal delivery in 69 cases and cesarean section in 51 cases. Perinatal complications included intrauterine distress in 21 cases, umbilical cord abnor malities in 13 cases, premature rupture of membranes in 17 cases, and meconium-stained amniotic fluid in 29 cases. Forty-two neonates required invasive mechanical ventilation.

### Grouping

Neonatal AKI was diagnosed according to the modified KDIGO criteria [9], and staged as follows:

Stage I: Serum creatinine (Scr) increased by 0.3 mg/dL within 48 hours, or increased to 1.5–1.9 times the previous baseline within 7 days, with urine output <1.0 mL/kg/h for more than 24 hours;

Stage II: Scr reached 2–2.9 times the previous baseline, with urine output <0.5 mL/kg/h for more than 24 hours;

Stage III: Scr 2.5 mg/dL or Scr increased to more than 3 times the previous baseline, or kidney replacement therapy was required clinically, with urine output <0.3 mL/kg/h for more than 24 hours.

Neonates were divided into AKI and non-AKI groups based on whether AKI occurred within 7 days after asphyxia.

### Methods

### Collection of general information

Clinical data on neonates’ gender, gestational age, birth weight, delivery method, perinatal complications, and invasive mechanical ventilation were collected.

### Laboratory indicators

Within 24 hours after birth, 2 mL of venous blood, 4–8 mL of midstream clean urine, and 0.5 mL of umbilical artery blood were collected. The venous blood and urine were centrifuged to collect the supernatant for testing. Serum creatinine (Scr) and blood urea nitrogen (BUN) levels were measured using an automatic biochemical analyzer (ADVIA 2400, Siemens, Germany). Plasma Angiotensin-I (Ang-I) and Angiotensin-II (Ang-II) levels were measured by chemiluminescence, urinary γ-glutamyl transferase (γ-GT) levels were measured using the diazo colorimetric method, and blood lactate levels were detected using an automatic blood gas analyzer.

### Renal ultrasound examination

After the neonatal hemodynamics stabilized, renal ultrasound was performed using a color Doppler ultrasound device (CX50, Philips, Netherlands) with a 1–5 MHz convex array probe for color Doppler flow imaging. Pulsed Doppler mode was used to sample the interlobar artery. Peak systolic and end-diastolic velocities were measured, and the renal interlobar resistance index (RRI) was calculated.

### Statistical analysis

Data were analyzed using SPSS 27.0 software. Measurement data were expressed as mean ± standard deviation (±s) and compared between groups using t-tests. Categorical data were expressed as n (%), and the chi-square test was used. Rank data were analyzed using the rank sum test. Correlation analysis was performed using Pearson’s method. Receiver operating characteristic (ROC) curves were used to analyze the diagnostic value of the study indicators for AKI after neonatal asphyxia. A P value < 0.05 was considered statistically significant.

## Results

### Incidence of AKI in neonates with different degrees of asphyxia

Among the 120 neonates with asphyxia, 40 had severe asphyxia and 80 had mild asphyxia. The overall incidence of AKI after neonatal asphyxia was 39.17%. The AKI incidence was significantly higher in the severe asphyxia group (62.50%) compared to the mild asphyxia group (27.50%) (P < 0.05) (Table 1).

**Table 1:** Comparison of AKI Incidence in Neonates with Different Degrees of Asphyxia (n (%)).

Degree of Asphyxia	AKI	Non-AKI	Total	χ^2^	P
Severe Asphyxia	25 (62.50)	15 (37.50)	40	13.710	<0.001
Mild Asphyxia	22 (27.50)	58 (72.50)	80		
**Total**	**47 (39.17)**	**73 (60.83)**	**120**		

### General information of neonates with asphyxia

There were no significant differences in the general information between the two groups of neonates (P > 0.05) (Table 2).

**Table 2:** Comparison of General Information between AKI Group and Non-AKI Group Neonates (n (%), ±s).

Item		AKI (n=47)	Non-AKI (n=73)	t/χ^2^	P
Gender	Male	25 (53.19)	46 (63.01)	1.142	0.285
	Female	22 (46.81)	27 (36.99)		
Gestational Age (weeks)		39.28±1.23	39.46±1.21	0.790	0.431
Birth Weight (g)		3329.46±327.13	3371.04±351.86	0.649	0.517
Delivery Method<br>(Mother)	Vaginal Delivery	29 (61.70)	40 (54.79)	0.558	0.455
	Cesarean Section	18 (38.30)	33 (45.21)		
Perinatal Complications	Intrauterine Distress	10 (21.28)	11 (15.07)	0.763	0.382
	Umbilical Cord Abnormalities	7 (14.89)	6 (8.22)	1.319	0.251
	Premature Rupture<br>of Membranes	9 (19.15)	8 (10.96)	1.577	0.209
	Meconium-Stained<br>Amniotic Fluid	14 (29.79)	15 (20.55)	1.332	0.249
Invasive Mechanical<br>Ventilation	Yes	20 (42.55)	21 (28.77)	2.416	0.120
	No	27 (57.45)	52 (71.23)		

### Laboratory indicators and renal ultrasound results in neonates with asphyxia

The levels of serum creatinine (Scr), blood urea nitrogen (BUN), and Angiotensin-II (Ang-II) in the AKI group were higher than those in the non-AKI group. Additionally, urinary γ-glutamyl transferase (γ-GT), blood lactate, and renal resistance index (RRI) were also significantly higher in the AKI group compared to the non-AKI group (P < 0.05). However, there was no significant difference in Angiotensin-I (Ang-I) levels between the two groups (P > 0.05) (Table 3).

**Table 3:** Comparison of Laboratory Indicators and Renal Ultrasound Results Between AKI and Non-AKI Groups (n (%), ±s). Note: Scr: Serum Creatinine. BUN: Blood Urea Nitrogen.

Item	AKI (n=47)	Non-AKI (n=73)	t/χ^2^	P
Scr (μmol/L)	62.46±10.29	57.63±11.82	2.296	0.023
BUN (mmol/L)	6.29±1.83	5.61±1.46	2.252	0.026
Ang-I (ng/mL)	5.91±1.43	6.58±2.16	1.877	0.063
Ang-II (pg/mL)	27.65±2.81	24.13±2.59	7.029	<0.001
Urinary γ-GT (U/L)	21.52±7.86	16.83±6.59	3.526	0.001
Blood Lactate (mmol/L)	5.63±0.97	4.49±0.83	6.871	<0.001
RRI	0.69±0.06	0.63±0.05	5.928	<0.001

### Correlation between blood angiotensin-II (Ang-II), urinary γ-GT, blood lactate, RRI, and Scr, BUN in neonates with asphyxia

Correlation analysis results showed that Ang-II, blood lactate, and RRI were positively correlated with serum creatinine (Scr) and blood urea nitrogen (BUN) (P < 0.05) (Table 4).

**Table 4:** Correlation Between Ang-II, Urinary γ-GT, Blood Lactate, RRI and Scr, BUN in Neonates with Asphyxia. Ang-II: Angiotensin II. RRI: Renal Resistive Index.

Group	Scr		BUN	
	*r*	*P*	*r*	*P*
Ang-II	0.455	<0.001	0.368	<0.001
Urinary γ-GT	0.522	<0.001	0.517	<0.001
Blood Lactate	0.466	<0.001	0.420	<0.001
RRI	0.573	<0.001	0.502	<0.001

### Analysis of risk factors for AKI after neonatal asphyxia

Indicators that showed statistical differences between the AKI and non-AKI groups were included in a multivariate logistic regression model. The results indicated that Ang-II, urinary γ-GT, blood lactate, and RRI are risk factors for the development of AKI after neonatal asphyxia (P < 0.05) (Table 5).

**Table 5:** Multivariate Logistic Regression Analysis of Risk Factors for AKI After Neonatal Asphyxia. Note: Scr: Serum Creatinine. BUN: Blood Urea Nitrogen. Ang-II: Angiotensin II. RRI: Renal Resistive Index.

Variables	*β*	*SE*	Wald χ^2^	*P*	*OR (95%CI)*
Scr	0.088	0.058	2.302	0.130	1.092 (0.975~1.223)
BUN	0.062	0.204	0.091	0.763	1.063 (0.13~1.587)
Ang-II	0.616	0.172	12.758	<0.001	1.852 (1.321~2.596)
Urinary γ-GT	0.096	0.043	5.120	0.024	1.101 (1.013~1.197)
Blood Lactate	1.047	0.381	7.552	0.006	2.849 (1.350~6.012)
RRI	1.069	0.419	6.509	0.011	2.912 (1.281~6.621)

### Diagnostic value of Ang-II, urinary γ-GT, blood lactate, and RRI for AKI after neonatal asphyxia

**Figure 1:**
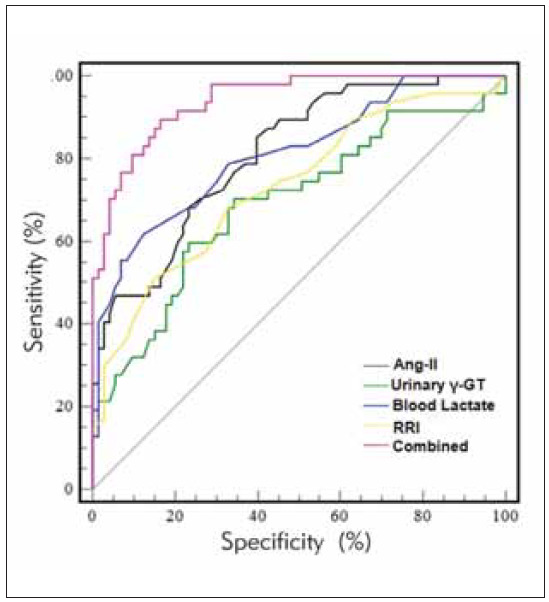
ROC Curve for the Diagnosis of AKI After Neonatal Asphyxia Using Ang-II, Urinary γ-GT, Blood Lactate, RRI, and Their Combined Application.

The area under the curve (AUC) for Ang-II in diagnosing AKI after neonatal asphyxia was 0.812, for urinary γ-GT was 0.693, for blood lactate was 0.811, and for RRI was 0.733. There were no significant differences in the AUC values between the individual indicators (P > 0.05). The AUC for the combined use of all four indicators was 0.941, which was higher than that of each individual indicator (z = 3.647, 5.058, 3.714, 4.496, all P < 0.001). For detailed information, see Table 6 and Figure 1.

**Table 6:** Diagnostic Value of Ang-II, Urinary γ-GT, Blood Lactate, RRI, and Their Combined Use for AKI After Neonatal Asphyxia. Note: Ang-II: Angiotensin II. RRI: Renal Resistive Index.

Indicator	AUC	95% *CI*	P Value	Cut-off Value	Youden Index	Sensitivity (%)	Specificity (%)
Ang-II	0.812	0.735~0.888	<0.001	25.0	0.454	85.11	60.27
Urinary γ-GT	0.693	0.592~0.794	<0.001	20.8	0.363	59.57	76.71
Blood Lactate	0.811	0.730~0.891	<0.001	5.2	0.494	61.70	87.67
RRI	0.733	0.639~0.827	<0.001	0.68	0.360	51.06	84.93
Combined	0.941	0.903~0.979	<0.001	-	0.729	89.36	83.56

## Discussion

AKI is a common complication after neonatal asphyxia, which significantly increases the neonatalmortality rate during hospitalization and is associated with adverse outcomes such as chronic kidney disease, negatively affecting the quality of life of the affected children [10]. The occurrence of AKI after neonatal asphyxia is related to the increased secretion of vasoactive substances, insufficient renal perfusion, and other factors. As the degree of asphyxia increases, the blood flow to the renal tissue decreases further, causing damage to the glomeruli and renal tubules, significantly increasing the risk of AKI. This study found that the incidence of AKI in neonates with severe asphyxia was significantly higher than in those with mild asphyxia, suggesting that severe asphyxia may increase the incidence of AKI. SCr, BUN, and urine output changes are important indicators reflecting renal function impairment. However, early symptoms in neonates are atypical, and the levels of SCr and BUN are influenced by many factors. Additionally, the applicability of KDIGO standards in neonates has not been fully established, which affects the diagnostic accuracy of neonatal AKI.

Angiotensin (Ang) is an endothelial growth factor that includes Ang-I and Ang-II, both of which play an important role in maintaining the integrity of the renal vascular endothelial barrier [11]. In this study, compared with the non-AKI group, the Ang-II levels in the AKI group were elevated, and the level of Ang-II was positively correlated with SCr and BUN, indicating that elevated Ang-II expression in neonates with asphyxia combined with AKI is closely related to renal function indicators. During the early stages of renal injury in neonates with asphyxia, a large number of inflammatory mediators and cytokines are released, which increase the excitability of the reninangiotensin system (RAS), leading to increased release of Ang-II. Ang-II causes sustained excessive constriction of the renal arteries, further worsening renal hypoperfusion, and progressing to AKI [12]. Abd El Wahab et al. [13] found that elevated Ang-II levels were associated with the occurrence of AKI and poor prognosis in patients with cirrhosis. In this study, there was no significant difference in Ang-I levels between the two groups, suggesting that RAS primarily regulates through the renin-Ang-II-Ang-II receptor 1 biological axis to maintain the normal function of the system. Gamma-glutamyl transferase (γ-GT) is an enzyme secreted by renal tubular epithelial cells, and its elevated levels in urine indicate renal tubular injury [14]. The results of this study showed that urinary γ-GT levels in the AKI group were significantly higher than those in the non-AKI group, indicating that urinary γ-GT levels can be elevated in the early stages of renal damage in neonates with asphyxia. The reason may be that the ischemic and hypoxic injury caused by asphyxia primarily affects the proximal convoluted tubules in the early stages of renal injury. γ-GT is mainly distributed in the cortical proximal tubule cells, and when these cells are damaged, the release of γ-GT into the urine increases significantly [15]. The correlation analysis in this study showed that urinary γ-GT was positively correlated with SCr and BUN, and logistic regression analysis confirmed that urinary γ-GT is an independent risk factor for AKI, further indicating that changes in urinary γ-GT levels are related to AKI in neonates with asphyxia. In this study, no significant differences were observed in gestational age and birth weight between the two groups of neonates (P > 0.05), suggesting that these factors may not be primary contributors to AKI occurrence in this study. However, future research could further explore specific high-risk subgroups.

Blood lactate is an indicator reflecting the hypoxic state of tissue cells. Its levels rise earlier than other monitoring indicators such as urine output and blood pressure [16]. Neonates with asphyxia cannot establish normal breathing, which induces microcirculatory disorders under hypoxic conditions, leading to the production of large amounts of lactate. In this study, blood lactate levels were significantly higher in the AKI group compared to the non-AKI group, and the expression levels were positively correlated with SCr and BUN. This suggests that blood lactate levels are associated with kidney function and the occurrence of AKI in neonates with asphyxia. The kidneys are rich in mitochondria, which play an important role in filtration and reabsorption, requiring mitochondrial energy support. In neonates with asphyxia, lactate accumulation causes microcirculatory disorders, which can lead to mitochondrial dysfunction in renal tubular epithelial cells by mediating excessive mitochondrial fission through lactylation of lysine 20 of the mitochondrial fission protein 1, thus resulting in AKI [17]. Matsushita et al. [18] also found that blood lactate levels in neonates were related to AKI and renal replacement therapy. Renal hemodynamic changes can reflect the status of renal blood flow and microcirculatory disorders. The renal resistance index (RRI) is associated with changes in renal blood flow and can be used to predict AKI in septic patients [19]. In this study, the AKI group had a higher RRI compared to the non-AKI group, and correlation and logistic regression analyses showed that RRI was related to AKI in neonates with asphyxia. These results are similar to those of El-Sadek et al. [20], suggesting that RRI can be used as an indicator to assess neonatal AKI.

ROC analysis in this study found that Ang-II, urinary γ-GT, blood lactate, and RRI all have diagnostic value for AKI after neonatal asphyxia. However, there were no significant differences in the AUC values between these indicators. Further combined diagnostic analysis showed that the AUC for the combined use of these indicators was 0.941, suggesting that combined testing may enhance the diagnostic performance for AKI in neonates with asphyxia through complementary multi-dimensional information.

In conclusion, changes in blood Ang-II, urinary γ-GT, blood lactate, and RRI levels after neonatal asphyxia are associated with the occurrence of AKI. These indicators can be used as biomarkers for the clinical diagnosis of AKI after neonatal asphyxia. Compared to individual indicators, combined diagnosis can further improve the diagnostic performance for AKI, offering valuable clinical reference.

## Dodatak

### Conflict of interest statement

All the authors declare that they have no conflict of interest in this work.
